# Knowledge of Emergency Management of Traumatized Teeth among Schoolteachers in Mashhad, Iran

**DOI:** 10.15171/joddd.2015.024

**Published:** 2015-06-10

**Authors:** Maryam Mehrabkhani, Behjatolmolok Ajami, Iman Parisay, Ali Bolboli, Golsa Akbarian

**Affiliations:** ^1^Assistant Professor of Pediatric Dentistry, Dental Material Research Center, School of Dentistry, Mashhad University of Medical Sciences, Mashhad, Iran; ^2^Professor of Pediatric Dentistry, School of Dentistry, Mashhad University of Medical Sciences, Mashhad, Iran; ^3^Dentist, Private Practice, Mashhad, Iran

**Keywords:** Dental trauma, knowledge, teacher

## Abstract

***Background and aims.*** Dental trauma is an important oral health problem in children that requires immediate and appropriate management for long term success. The aim of this study was to evaluate the knowledge of elementary school teachers about emergency management of traumatic dental injuries in children.

***Materials and methods.*** A total of 163 teachers from 21 elementary schools from Mashhad, Iran participated in this study. Data were collected using a two-part questionnaire comprised of questions regarding demographic data and participants' knowledge about dental trauma.

***Results. ***The level of the teachers' knowledge was moderate (53.3%). Of the 163 participants, 104 (63.8%) were females and 59 (36.2%) were males. Among several predictors that were surveyed in this study, only the age of respondents signifi-cantly impacted the teachers' knowledge (P = 0.004).

***Conclusion.*** The results of this study showed the moderate level of knowledge of teachers regarding emergency manage-ment of traumatized teeth. Adding dental trauma management courses in the teacher training curriculum and requiring teachers to be updated in regards to this issue can improve their knowledge and thus led to better management of traumatic dental injuries in children.

## Introduction

Dental trauma is an important and prevalent oral health problem in children that may cause pain and distress.^1^ Of those children who have experienced such a trauma, about thirty percent have sustained injuries to their primary dentition and twenty-two percent to their permanent dentition.^2^ Most traumatic dental injuries occur in children between the ages of eight and eleven,^3^ and the majority have occurred at home followed by school.^4,5^

In order to have long-term success, the management or treatment of traumatized teeth should be accomplished immediately;^1^ however, many studies have presented that treatment needs of traumatic dental injuries are not properly met. In Finland only 25% and in the United Kingdom only 10-15% of children with traumatized teeth had received proper treatment.^6^ The time between the trauma occurrence and receiving dental management ranged between 2 hours and 3 years.^6^

Most of the time first aid is given by lay people such as parents, teachers or coaches; hence, their knowledge of emergency management of traumatized teeth is critical for long term success. Unfortunately, many studies have reported lack of knowledge in this first-level care population.^6-9^ In Jordan, possibly due to people's lack of knowledge, lots of traumatic dental injuries in children have been neglected, resulting in unfavorable long-term prognosis.^4,7^

The purpose of this study was to assess the level of knowledge of school teachers in Mashhad, Iran regarding emergency care of dental injuries by use of a two-part questionnaire.

## Material and Methods

This cross-sectional study was carried out in Mashhad, Iran in March 2009, with the targeted population being elementary school teachers. One-hundred and sixty three teachers from randomly selected elementary schools participated in this survey.

All participants signed an informed consent form and were assured of strict confidentiality. The questionnaire was translated and designed from a study done by Chan et al.^9^ Reliability of the questionnaire was assigned by the Cronbach’s alpha method to measure the internal consistency among the survey items (Cranbach’s alpha=0.81). Next, the copies of the questionnaire were distributed to a group of teachers in order to determine how well the questionnaire was composed and, if necessary, it was corrected prior to administering the questionnaire to the study population. The questionnaire was approved by the Ethical Committee of the Mashhad University of Medical Sciences.

The questionnaire consisted of two parts (see Additional File 1). Part I had six questions regarding personal and professional data of the teachers including age, sex, educational level, and their first aid training background.

Part II had 16 multiple choice questions, in which the first three questions were about the management of two imaginary cases of traumatized teeth.

The first case depicted a mild incident of crown fracture and the second case depicted a severe scenario that involved an avulsion.

The questionnaires were filled out by teachers at their respective schools. Afterwards, all of the questionnaires were collected and analyzed. Results were expressed as a number and percentage of correct answers of respondents.

To evaluate the range of the teachers' knowledge, the correct answers were counted and the percentages of correct answers were determined (<50% poor; 50-75% moderate; >75% good).

The results of the questionnaire were analyzed by the frequency distribution and the Kruskal-Wallis and independent *t*-tests were used to evaluate the teachers’ knowledge. The level of significance was set at P = 0.05. Statistics were analyzed by the SPSS software version 11.5 (Chicago, IL, USA).

## Results

Seven randomly selected districts of Mashhad, Iran participated in our study and a total of 163 questionnaires in 21 elementary schools were collected from teachers. One-hundred and four (63.8%) were females and 59 (36.2%) were males. Of the total respondents, 72 (44.1%) were 30-39 years old and 77 (47.2%) had Bachelor degrees (Table 1).

**Table 1:** Demographic characteristics of the teachers

**Demographic information**		**N**	**%**
**Gender**	Female	104	**63.8**
	Male	59	**36.2**
**Age(Year)**	20-29	64	**39.2**
	30-39	72	**44.1**
	40-49	22	**13.4**
	50 or above	5	**3.3**
**Educational level**	Diploma	4	**2.5**
	College	73	**44.8**
	Bachelors' degree	77	**47.2**
	Master of science or above	9	**5.5**

Seven (4.3%) of the respondents were health teachers, 55 (33.7%) were able to remember receiving first aid training and of these 55, only four (2.5%) claimed that the training course included first aid for traumatized teeth. Hence, the knowledge of teachers toward emergency management of traumatized teeth was moderate (53.3%).

The result from the independent t-test showed that there was not any significant difference in the number of correct answers about the knowledge of teachers in regards to emergency management of traumatized teeth in relation to sex (P = 0.385; Table 2).

**Table 2:** The average score of correct answer in relation to age, sex, educational level, attending first aid training, dental trauma training courses and being school health teacher

**Variable**		**Average Score of correct answer**	**P- Value**
**Gender**	Female	8.42±2.25	0.385
	Male	8.75±2.3	
**School health teacher**	Yes	10.14±3.48	0.056
	No	8.47±2.19	
**First aid training**	Yes	8.67±2.74	0.632
	No	8.47±1.99	
**Dental trauma management training**	Yes	10.25±2.75	0.293
	No	8.5±2.25	
**Educational level**	Diploma	9.25±1.7	0.674
	College	8.68±2.35	
	Bachelor's degree	8.35±2.25	
	Master of science or higher education	8.67±2.06	
**Age (year)**	20-29	7.77±2.18	0.004^*^
	30-39	8.88±2.06	
	40-49	9.33±2.30	
	50 or above	10.4±3.57	
^*^Statistical Significant			

The Crustal-Wallis test showed that there was no significant difference in the number of correct answers in relation to the level of education (P = 0.674), but there was significant difference in relation to age (P = 0.004; Table 2).

The independent t-test showed that there was no significant difference between teachers, who attended a first aid training course that included dental trauma management (P = 0.632 & P = 0.293, respectively; Table 2).

Furthermore, there was no significant difference between health teachers and the other teachers (P = 0.056; Table 2).

Part II of the questionnaire was related to the knowledge of teachers regarding dental management of traumatized teeth. The average score for all respondents was 53.3%. The percentage of response to each question of the questionnaire is presented in Table 3.

**Table 3:** The percentage of correct answers to questions of questionnaire

**Question number**	**Correct answer**	
	**N**	**%**
**Q**_1_	73	44.8
**Q**_ 2_	105	64.4
**Q**_ 3_	77	47.2
**Q**_ 4_	103	63.2
**Q**_ 5_	108	66.3
**Q**_ 6_	22	50.3
**Q**_ 7_	134	82.2
**Q**_ 8_	17	10.4
**Q**_ 9_	140	85.9
**Q**_ 10_	125	76.7
**Q**_ 11_	59	36.2
**Q**_ 12_	100	61.3
**Q**_ 13_	58	61.3
**Q**_ 14_	96	58.9
**Q**_ 15_	59	36.2
**Q**_ 16_	54	33.1

## Discussion

The level of knowledge of dental trauma and emergency management as shown in this survey was moderate. This result indicates a lack of awareness regarding the importance of early and optimal treatment of dental traumas among elementary schoolteachers.

The level of knowledge among teachers who participated in a first aid training course that included dental trauma management was higher than those teachers who did not participate in such a course. Although this difference was not statistically significant, it shows that participating in a dental trauma management course and a continuing education program regarding first aid dental trauma management training might improve the knowledge and skills necessary in managing a dental trauma. Moreover, a higher level of knowledge was found among teachers older than 50 years, which can be due to having more experiences compared to those of their younger counterparts. 

The two imaginary cases in second part of the questionnaire were designed to evaluate the school teachers’ general knowledge of two different types of traumatic dental injuries. The ages of the injured children in these cases were intentionally selected as 8 and 13 respectively, because children between these ages have a greater risk of sports related dental injuries.^10^

In case 1, only 44.8% of the teachers recognized that a traumatized incisor belonged to the permanent dentition of the 8-year-old girl. This indicates that general dental knowledge of the surveyed teachers was inadequate, similar to the results of Chan et al^9^ (46.8%) and slightly better than the results reported by Al-Jundi et al.^7^

In case I, the general knowledge of appropriate action was found to be moderate (43.7%), which was in contrast to the study of Al-Jundi et al,^7^ but similar to the study of Chan et al.^9^ In this study, 64.4% of the teachers answered correctly by opting to contact the parents and advising them to have their child seen by the dentist promptly. This moderate level of knowledge indicates the need for continuous dental emergency training for schoolteachers. These educational programs should be provided to all schoolteachers regardless of their educational level or gender, as the results of the present study indicates no difference in the level of knowledge with regards to these variables.

In case II, which was about the emergency approach to a maxillary permanent avulsed incisor in an 11-year-old boy, only 47.2% of teachers answered correctly and opted to put the tooth back into the socket immediately. This figure is still higher than those reported by Fux-Noy et al (4.3%),^6^ Chan et al (5.4%),^9^ Hamilton et al (18.3%),^11^ Pacheco et al (18.3%),^12^ and Blakytny et al (25.5%).^13^

In this study, 63.2% of the respondents indicated that they would contact and refer a child to a dentist immediately after dental trauma. In other words, more than one-thirds of the respondents would fail to do what is necessary in a real-life situation. The same is true of a previous study with the figure reported 60.4%.^6^

In this survey, 66.3% of respondents chose a tetanus vaccine injection as a proper action after a contaminating damage to a tooth, which indicates a fair level of knowledge.

This study found 85.9% of the teachers would contact a dentist in case of dental trauma, which is a similar to the study of Fux-Noy et al.^6^ Different figures have been reported by Chan et al (48.8%),^9^ Hamilton et al (53.8%),^11^ and Mori et al (60.6%).^14^

In this study, 76.7% of the respondents were aware of the fact that an avulsed tooth should be replanted immediately, a similar figure to that of Hashim.^15^ Also, a moderate level of 61.3% considered replantation of an avulsed primary tooth as contraindicated. The knowledge of the respondents toward washing a contaminated avulsed tooth was poor and only 36.2% answered correctly and chose milk and water for washing a tooth. Only 33.1% chose milk as the best storage media for an avulsed tooth. This is similar to another study where 34% of respondents chose milk and 32% chose HBSS.^16^ A study by Blakytny et al^13^ found that 45.6% of the teachers chose milk as the most suitable media; in this instance the percentage of correct answers in our study is very high in comparison with other studies among teachers.^9,11,17^ However, the overall knowledge of dental trauma management among schoolteachers was poor, and thus, training on this subject is necessary to improve the situation.

Teacher training should include first aid courses that include emergency dental trauma management, posters at schools and playgrounds, and media campaigns. Literature has shown that those who knew what to do for an avulsed permanent tooth had received this information from a variety of sources including posters at dental clinics or health centers, articles in newspapers or professional journals, and by word of mouth.^6^ Al-Asfour et al^18^ observed an improvement in the knowledge of teachers after an informative 30-min lecture about tooth avulsion.^18^

## Conclusion

The knowledge of traumatic dental injuries management among elementary schoolteachers in Mashhad is in a moderate level. Adding dental trauma management courses to the curriculum of teacher training programs and informing teachers on a regular basis through various methods deems necessary. Moreover, the teachers must be made aware of the importance of this issue and be motivated to seek more information on their own.

**Additional file 1. The questionnaire used for the study. This material is available online as a Word 97-2003 Document.**

## References

[1] Andreasen JO, Andreasen FM, Andersson L. Textbook and Color Atlas of Traumatic Injuries to the Teeth, 4th ed. Copenhagen: Munksgaard, 2007.

[2] Andreasen JO, Rovn JJ (1972). Epidemiology of traumatic dental injuries to primary and permanent teeth in a Danish population simple. Int J Oral Surg.

[3] Caglar E, Ferriera LP, Kargul B (2005). Dental trauma management knowledge among a group of teachers in two south European cities. Dent Traumatol.

[4] Al-Jundi SH (2002). Dental emergencies presenting to a dental teaching hospital due to complications from traumatic dental injuries. Dental Traumatol.

[5] O' Neil DW, Clark MV, Lowe JW, Harrington MS (1989). Oral trauma in children a hospital survey. Oral Serg Oral Med Oral Pathol.

[6] Fux-Noy A, Samat H, Amir E (2011). Knowledge of elementary school teachers in Tel-Aviv, Israel, regarding emergency care of dental injuries. Dent Traumatol.

[7] Al-Jundi SH, Al-Waeilli H, Khairalah K (2005). Knowledge and attitude of Jordanian school hearth teachers with regards to emergency management of dental trauma. Dent Traumatol.

[8] Vergotine RJ, Govons R (2010). Public school educatior's knowledge of initial management of dental trauma. Dent Traumatol.

[9] Chan AW, Wang TK, Cheung GS (2001). Lay knowledge of physical health education teachers about the emergency management of dental trauma in Hong Knog. Dent Traumatol.

[10] Hayrinen-Immonen R, Sane J, Perkki K, Malmstrom M (1990). A six-year follow-up study of sports-related dental injuries in children and adolescents. Endod Dent Traumatol.

[11] Hamilton FA, Hill FJ, Mackie IC (1997). Investigation of lay knowledge of the management of avulsed permanent incisors. Endod Dent Traumatol.

[12] Pacheco LF, Filho PFG, Letra A, Menezes R, Villoria GEM, Ferreira SM (2003). Evaluation of the knowledge of the treatment of avulsion in elementary school teachers in Rio de Jonerio, Brazil. Dent Traumatol.

[13] Blakytny C, Surbnts C, Thomas A, Hunter ML (2001). Avulsed permanent incisors: knowledge and attitudes of primary school treatment with emergency management. Int Paediat Dent.

[14] Mori GG, Turcio KHL, Borro VPB, Mariusso AM (2007). Evaulation of the Knowledge of tooth avulsion of school professionals from Adamentina, Sau Paulo; Brazil. Dent Traumatol.

[15] Hashim R (2011). Dental trauma management awareness among primary school teachers in the Emirate of Ajemen, United Arab Emirates. Eur J Paediatr Dent.

[16] McIntyre JD, Lee JY, Trope M, Vann WF (2008). Elementary school staff knowledge about dental injury. Dent Traumatol.

[17] Sae-Lim V, Lim LP (2001). Dental trauma management awareness of Singapore preschool teachers. Dent Traumatol.

[18] Al-As Four A, Andersson L, Al-Jome Q (2008). School teacher’s knowledge of tooth avulsion and dental first aid before and after receiving information about avulsed teeth and replantation. Dent Traumatol.

